# Supplementary material for: Identification of photoperiod-induced specific miRNAs in the adrenal glands of Sunite sheep (Ovis aries)
Source: Front Vet Sci. 2022 Jul 22;9:888207. doi: 10.3389/fvets.2022.888207 (PMC9354845; doi:10.3389/fvets.2022.888207)
Supplement: Supplementary file 1 [file Data_Sheet_1.ZIP › Supplementary file/miRDeep2 file.zip!/miRDeep2 file/Novel_Structure/SPLP42A3/Novel_145.pdf]

Provisional ID	:	NC_019459.2_4604
Score total	:	236.4
Score for star read(s)	:	3.9
Score for read counts	:	229
Score for mfe	:	1.9
Score for randfold	:	1.6
Score for cons. seed	:	
Total read count	:	461
Mature read count	:	460
Loop read count	:	0
Star read count	:	1

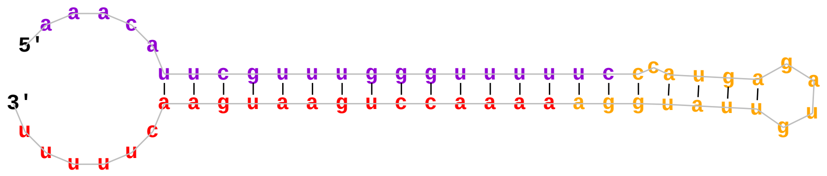

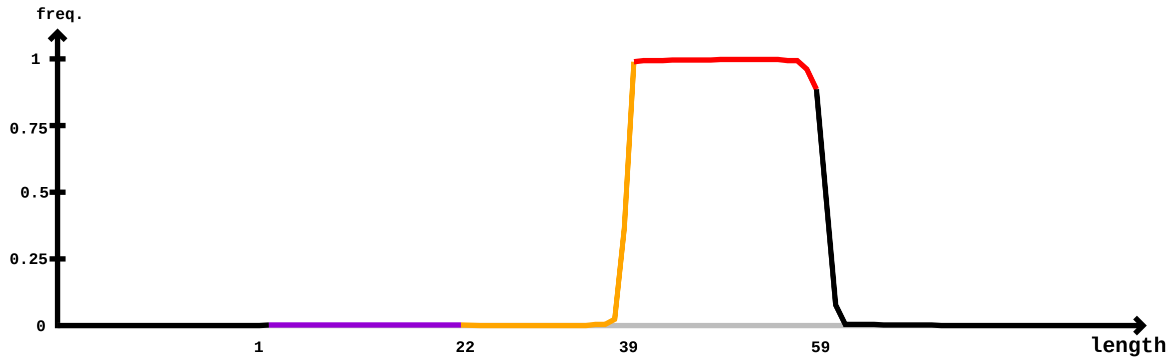

Star **Mature**

```
5'- uuuaaauauaggguuggacaaaacauucguuugggguuuuucccaugagauguuauggaaaaaccugaaugaacuuuuugaccaacccaauauuuuggguuuagagguuuaa   -3'   obs
    uuuaaauauaggguuggacaaaacauucguuugggguuuuucccaugagauguuauggaaaaaccugaaugaacuuuuugaccaacccaauauuuuggguuuagagguuuaa         exp
    ...((((((.(((((((.(((((..(((((((((((((((((.((((....)))))))))))))))))))))..))))).))))))).)))))).................    reads   mm   sample
    ....................aaacauucguuugggguuuuuc......................................................................    1       0    seq
    ......................................................uggaaaaaccCgaaugaacu.....................................    2       1    seq
    ........................................................gaaaaaccCgaaugaacuuu...................................    1       1    seq
    ........................................................gaaaaaccAgaaugaacuuuu..................................    1       1    seq
    ........................................................gaaaaaccugaaugaacuuuu..................................    4       0    seq
    ........................................................gaaaaaccCgaaugaacuuuu..................................    1       1    seq
    ........................................................gGaaaaccugaaugaacuuuuu.................................    1       1    seq
    ........................................................gaaaaaccugaaCgaacuuuuu.................................    1       1    seq
    .........................................................aaaaaccCgaaugaacuuu...................................    1       1    seq
    .........................................................aaaaaUcugaaugaacuuu...................................    12      1    seq
    .........................................................aaaaaccugaaugaacCuu...................................    1       1    seq
    .........................................................aaaaaUcugaaugaacuuuu..................................    26      1    seq
    .........................................................aaaaaccugaaGgaacuuuu..................................    1       1    seq
    .........................................................aaaaaccugaaugaacCuuuu.................................    1       1    seq
    .........................................................aaaaaUcugaaugaacuuuuu.................................    78      1    seq
    .........................................................Gaaaaccugaaugaacuuuuu.................................    1       1    seq
    .........................................................aaaaaccugaaugaacCuuuug................................    1       1    seq
    .........................................................aaaaaccCgaaugaacuuuuug................................    2       1    seq
    .........................................................aaaaaccugaaCgaacuuuuug................................    3       1    seq
    .........................................................aaaaaUcugaaugaacuuuuug................................    24      1    seq
    .........................................................aaaaaccugaauAaacuuuuuga...............................    1       1    seq
    .........................................................aaaaaccugaaugaacuuuuuga...............................    7       0    seq
    ..........................................................aaaaccugaaugGacuuuu..................................    1       1    seq
    ..........................................................aaaaccAgaaugaacuuuu..................................    1       1    seq
    ..........................................................aaaCccugaaugaacuuuuu.................................    1       1    seq
    ..........................................................aaaaccAgaaugaacuuuuu.................................    2       1    seq
    ..........................................................aaaaccugaaugaacuuuuu.................................    8       0    seq
    ..........................................................aaaaUcugaaugaacuuuuu.................................    247     1    seq
    ..........................................................aaaaccugaaugaacuuuuuU................................    2       1    seq
    ..........................................................Gaaaccugaaugaacuuuuug................................    1       1    seq
    ..........................................................aaaCccugaaugaacuuuuug................................    2       1    seq
    ..........................................................aaaaccugaaugaacuuuuuga...............................    23      0    seq
    ..........................................................aaaaccugaauAaacuuuuuga...............................    1       1    seq
```

```
                    Star                                    Mature
uuuaaauauaggguuggacaaaacauucguuuggguuuuucccaugagauguuauggaaaaaccugaaugaacuuuuugaccaacccaauauuuuggguuuagagguuuaa
..........................................................aaGaccugaaugaacuuuuuga...............................    1    1    seq
...........................................................aaaccAgaaugaacuuuuug................................    1    1    seq
...........................................................aaaAcugaaugaacuuuuuga...............................    1    1    seq
..............................................................ccugaaugaacuuuuugaccaa...........................    1    0    seq
...................................................................augaacuuuuugaccaacccaaA.....................    1    1    seq
```
